# 762 Resumption of Surgical Missions in Light of COVID-19: A Paradigm Shift

**DOI:** 10.1093/jbcr/irac012.315

**Published:** 2022-03-23

**Authors:** Robert J Dabek, Gennadiy Fuzaylov

**Affiliations:** Ascension St.Agnes Hospital, Baltimore, Maryland; Massachusetts General Hospital, Boston, Massachusetts

## Abstract

**Introduction:**

The COVID-19 pandemic has disrupted the lives of billions of people globally. Some medical systems continue to be overburdened due to the viral illness leading to incredible public health challenges domestically as well as abroad. However, with vaccination distribution increasing globally, many are pushing for a return to some form of normalcy. In the medical community, some are weighing the risks of returning to global health missions and considering protective strategies to minimize risk of viral spread.

**Methods:**

Here we describe our experience in returning to an annual burn reconstruction mission in a low- and middle-income country (LMIC).

**Results:**

We have implemented protective strategies and successfully carried out a return mission trip. Our team of 10 individuals was able to perform over 80 procedures on 26 pediatric patients in 4 operative days. There were no major complications reported.

**Conclusions:**

Protection of our team and our patients from the risk of COVID-19 infection was paramount given the high mortality rate and disease duration. We applied a variety of protective strategies and altered mission protocol to limit exposure and transmission. The primary modifications (including; eliminating day of clinic with increased utilization of telemedicine for preoperative screening, only one vaccinated care giver permitted in the hospital, COVID-19 pre-operative screening for parents and patients, and increasing operative complexity) are likely to remain in place for the duration of the pandemic.

